# Gastric cancer risk in relation to Helicobacter pylori infection and subtypes of intestinal metaplasia.

**DOI:** 10.1038/bjc.1998.453

**Published:** 1998-07

**Authors:** M. S. Wu, C. T. Shun, W. C. Lee, C. J. Chen, H. P. Wang, W. J. Lee, J. T. Lin

**Affiliations:** Department of Internal Medicine, National Taiwan University Hospital, Taipei.

## Abstract

Helicobacter pylori (H. pylori) infection and intestinal metaplasia (IM) are each associated with an increased risk of gastric cancer (GC). To explore further the influences of H. pylori and IM on GC, H. pylori and subtypes of IM were evaluated in 135 sex and age-matched case and control pairs. Odds ratios (ORs) with 95% confidence intervals of developing GC were calculated for each risk factor using multiple logistic regression analysis. ORs for H. pylori infection and IM were 2.43 (1.29-4.65) and 4.59 (2.58-8.16), respectively, and those for different IM subtypes gave values of 0.82 (0.28-2.36) for type I, 2.03 (0.95-4.34) for type II and 39.75 (14.34-110.2) for type III. Stratification analysis by histological subtype and stage of GC showed a particularly high OR for IM in intestinal type (12.8, 4.73-34.83) and early GC (6.40, 2.25-18.18). Our data indicate that both H. pylori and IM are related to GC risk. Type III IM is a more specific marker of premalignancy, with relevance, in particular, to the early and intestinal type of GC.


British Journal of Cancer (1998) 78(1), 125–128
© 1998 Cancer Research Campaign

### Gastric cancer risk in relation to *Helicobacter pylori* infection and subtypes of intestinal metaplasia

**M-S Wu[1], C-T Shun[2], W-C Lee[3], C-J Chen[3], H-P Wang[4], W-J Lee[5], J-C Sheu[1] and J-T Lin[1]**

Departments of [1]Internal Medicine and [2]Pathology, National Taiwan University Hospital, Taiwan; [3]Graduate Institute of Epidemiology, College of Public Health, National Taiwan University, Taiwan; Departments of [4]Emergency Medicine and [5]Surgery, National Taiwan University Hospital, Taiwan

**Summary** *Helicobacter pylori* (*H. pylori*) infection and intestinal metaplasia (IM) are each associated with an increased risk of gastric cancer (GC). To explore further the influences of *H. pylori* and IM on GC, *H. pylori* and subtypes of IM were evaluated in 135 sex and age-matched case and control pairs. Odds ratios (ORs) with 95% confidence intervals of developing GC were calculated for each risk factor using multiple logistic regression analysis. ORs for *H. pylori* infection and IM were 2.43 (1.29–4.65) and 4.59 (2.58–8.16), respectively, and those for different IM subtypes gave values of 0.82 (0.28–2.36) for type I, 2.03 (0.95–4.34) for type II and 39.75 (14.34–110.2) for type III. Stratification analysis by histological subtype and stage of GC showed a particularly high OR for IM in intestinal type (12.8, 4.73–34.83) and early GC (6.40, 2.25–18.18). Our data indicate that both *H. pylori* and IM are related to GC risk. Type III IM is a more specific marker of premalignancy, with relevance, in particular, to the early and intestinal type of GC.

**Keywords:** gastric cancer; *Helicobacter pylori*; intestinal metaplasia; odds ratio

Gastric cancer (GC) is one of the commonest cancers in the world (Parkin et al, 1993) as well as in Taiwan (Wu et al, 1994). Despite some improvement in its treatment, the 5-year survival rate of GC remains low. Therefore, further exploration of biological features and causes of GC is needed to reduce its occurrence. Both environmental and genetic factors are crucial in the multistage model of gastric tumorigenesis (Correa, 1992). In sequential changes from superficial gastritis to dysplasia and cancer, intestinal metaplasia (IM) plays a pivotal role. This is testified by a high frequency of IM in patients with GC and in their relatives, a similar topographic distribution of IM and GC, and a high IM occurrence in endemic areas with a high risk of GC (Dobrilla et al, 1994). Although most investigators agree with Stemmermann (1994) that the risk of GC is proportional to the extent of IM and although IM has been intensively explored as a possible premalignant lesion of GC, controversy exists because of many confounding factors in previous retrospective studies (Antonioli, 1994). Because of the heterogeneous nature of IM, a recent interest has been focused on the interrelationship between GC and different subtypes of IM (Jass et al, 1979; Turani et al, 1986; Filipe et al, 1994).

In recent years, abundant epidemiological data have consistently documented a higher rate of *H. pylori* infection in GC (Forman et al, 1991; Nomura et al, 1991; Parsonnet et al, 1991). *H. pylori*-induced inflammation may facilitate gastric carcinogenesis by increasing the rate of cell replication, decreasing the concentration of ascorbic acid and inducing DNA damage via reactive oxygen species (Sobala et al, 1993; Bechi et al, 1996). It is worth noting that the prevalence of IM is also closely related to *H. pylori* infection (Rugge et al, 1996). Furthermore, chronic gastritis due to *H. pylori* infection may progress to IM and even GC (Correa, 1992; Dobrilla et al, 1994; Stemmermann, 1994). Nevertheless, few studies are available to redefine the interactions of these two factors with respect to gastric carcinogenesis. A preliminary observation has suggested that both IM and *H. pylori* infection are important targets for prevention of GC (Caselli, 1996). We undertook this study to investigate the relations of *H. pylori* and of IM and the risk of GC.

*Received 12 September 1997*
*Revised 5 January 1998*
*Accepted 12 January 1998*

*Correspondence to:* J-T Lin, Department of Internal Medicine, National Taiwan University Hospital, No. 7 Chung-Shan S. Rd., Taipei, Taiwan

### MATERIALS AND METHODS

### Patients and tissues

With their informed consent, a total of 160 patients with histologically diagnosed GC were recruited consecutively for study from January 1995 to January 1997. None of them received chemotherapy before surgery. Tumours were classified as intestinal or diffuse subtypes according to the Lauren criteria (Lauren, 1965). The extent of tumour invasion was further divided into early or advanced GC according to the criteria proposed by the Japanese Research Society for Gastric Cancer (Murakami, 1971). After excluding 25 patients with mixed or undetermined histological subtypes of GC in gastrectomy specimens, 135 patients were finally included in this study. Tumorous, adjacent and non-tumorous portions were resected from surgical specimens and stored for further examination. Controls matched one-to-one with cases on age (within 3 years), sex, ethnic group and residential area were also selected from subjects receiving endoscopic examinations that did not reveal any ulcer nor tumour in the stomach. During this procedure, five specimens were biopsied including two from the lesser curvature of the antrum, one from the incisura angularis and two from the lesser curvature of the corpus of the stomach.

**Table 1** Frequency distribution of age, gender, ethnic group, *H. pylori* infection and intestinal metaplasia of 135 matched pairs of gastric cancer cases and controls

| Variable | Cases n (%) | Control n (%) |
|---|---|---|
| Age (year) | | |
| ≤ 40 | 17 (12.6) | 18 (13.3) |
| 41–50 | 21 (15.6) | 19 (14.1) |
| 51–60 | 23 (17.0) | 24 (17.8) |
| 61–70 | 37 (27.4) | 37 (27.4) |
| > 70 | 37 (27.4) | 37 (27.4) |
| Gender | | |
| Male | 71 (52.6) | 71 (52.6) |
| Female | 64 (47.4) | 64 (47.4) |
| Ethnic group | | |
| Fukienese | 109 (80.7) | 109 (80.7) |
| Hakka | 10 (7.4) | 10 (7.4) |
| Other | 16 (11.9) | 16 (11.9) |
| *H. pylori*[a] | | |
| Negative | 49 (36.3) | 60 (44.4) |
| Positive | 86 (63.7) | 75 (55.6) |
| Intestinal metaplasia | | |
| Negative | 45 (33.3) | 86 (63.7) |
| Positive | 90 (66.7)[b] | 49 (36.3) |
| Type I | 6 (4.4) | 17 (12.6) |
| Type II | 22 (16.3) | 25 (18.5) |
| Type III | 62 (46.0) | 7 (5.2) |

[a]Serological diagnosis by IgG antibodies to *H. pylori*. [b]$P < 0.0001$ vs controls.

**Table 2** Histological features of diffuse and intestinal types of gastric cancer

| Features | Diffuse type ($n = 57$) | Intestinal type ($n = 78$) |
|---|---|---|
| Depth of invasion | | |
| Early | 24 (42.1)[a] | 23 (29.5) |
| Advanced | 33 (57.9) | 55 (70.5) |
| Adjacent metaplasia | | |
| Positive | 19 (33.3)[b] | 71 (91.0) |
| Negative | 38 (66.7) | 7 (9.0) |

[a]Numbers in parentheses are percentages. [b]$P < 0.0001$ between diffuse and intestinal type.

### Serological tests for *H. pylori*

An aliquot of 5 ml of heparinized blood was collected from each study subject, and the serum was separated on the same day and stored at –70°C until further testing. The titre of serum IgG antibody against *H. pylori* was determined using an enzyme-linked immunosorbent assay as previously described (Lin et al, 1993).

### Histopathological determination of intestinal metaplasia and its subtypes

Specimens from patients and controls were fixed in 10% buffered formalin, embedded in paraffin, sectioned and stained with haematoxylin and eosin (H&E). If IM was present in H&E staining, a further section was stained using the high iron diamine (HID)/alcian blue (AB) technique (Lev, 1965). The HID/AB stain differentiates acidic mucin into sialomucins (blue) and sulphomucins (brown-black). Using HID/AB and H&E staining, the metaplastic lesions were further classified into three subtypes: type I, i.e. complete IM characterized by resembling normal intestinal epithelium; type II, i.e. incomplete IM expressing sialomucins but not sulphomucins; and type III, i.e. incomplete IM expressing sulphomucins. If IM of more than one subtype was present in a given sample, the case was assigned to the least mature subtype, as proposed previously (Rugge et al, 1996).

**Table 3** Multivariate-adjusted odds ratios of developing gastric cancer for *H. pylori* infection and intestinal metaplasia subtypes

| Factors | Adjusted odds ratios | 95% Confidence intervals |
|---|---|---|
| *H. pylori* | | |
| Negative | 1.00 | Referent |
| Positive | 2.43 | 1.29–4.65 |
| Intestinal metaplasia | | |
| Negative | 1.00 | Referent |
| Positive | | |
| Type I | 0.82 | 0.28–2.36 |
| Type II | 2.03 | 0.95–4.34 |
| Type III | 39.75 | 14.34–110.20 |
| I+II+III | 4.59 | 2.58–8.16 |

### Statistical analysis

Odds ratios (ORs), i.e. estimators of relative risk, and the corresponding 95% confidence intervals (CI) of developing GC were calculated for each risk factor using multiple logistic regression analysis (Breslow and Day, 1980). Chi-square and Fisher's exact tests were used to analyse categorical data. A *P*-value less than 0.05 was considered to be statistically significant.

### RESULTS

The frequency distribution of age, gender, ethnic groups, *H. pylori* infection and IM of GC patients and their matched controls is shown in Table 1. The age distribution of GC patients in this study was similar to that previously reported in Taiwan (Wu et al, 1994). Most study subjects were Fukienese. The frequency of *H. pylori* infection was different between GC (63.7%) and controls (55.6%), and the ORs (2.43, 95% CI 1.29–4.65) for *H. pylori* of developing GC was significant after adjustment for IM. GC cases had a higher frequency of IM than controls (66.7% vs 36.3%, $P < 0.0001$).

The histological features of diffuse and intestinal type GC are summarized in Table 2. There were 57 patients with diffuse type GC and 78 with intestinal type GC. According to the depth of invasion, the frequency of early lesion in diffuse type GC (42.1%) was higher than that of intestinal type GC (29.5%, $P = 0.18$). Intestinal type GC had a significantly higher frequency of coexistent IM (91.0%) than diffuse type (33.3%, $P = 0.0001$).

As shown in Table 3, ORs for *H. pylori* and IM of developing GC were 2.43 (1.29–4.65) and 4.59 (2.58–8.16) respectively. A significantly increased risk of GC was noted for type III IM (OR 39.75; 95% CI 14.34–110.20). The relationship between *H. pylori* and GC after stratification by the presence or absence of IM is summarized in Table 4. Seropositivity of *H. pylori* was less frequent in type III IM (33 out of 62, 53.2%) than in type I plus

© Cancer Research Campaign 1998

**Table 4** The relationship between *Helicobacter pylori* seropositivity and gastric cancer after stratification by the presence or absence of intestinal metaplasia

| | Gastric cancer | | |
|---|---|---|---|
| | Total case number | *H. pylori* (+)[a] case number | Positive rate (%) |
| Intestinal metaplasia | | | |
| Negative | 45 | 31 | 68.9 |
| Positive | 90 | 55 | 61.1 |
| Type I | 6 | 5 | 83.3 |
| Type II | 22 | 17 | 77.3 |
| Type III | 62 | 33 | 53.2[c] |

[a]Positive IgG antibodies to *H. pylori*. [c]Type III vs type I + type II, $P = 0.04$

**Table 5** Multivariate-adjusted odds ratios of developing gastric cancer for intestinal metaplasia stratified by subtype and stage of gastric cancer

| Type/Stage | Adjusted odds ratio | 95% Confidence interval |
|---|---|---|
| Intestinal type | 12.83 | 4.73–34.83 |
| Diffuse type | 3.14 | 1.19–8.27 |
| Early stage | 6.40 | 2.25–18.18 |
| Advanced stage | 3.99 | 1.98–8.07 |

type II IM (22 out of 28, 78.6%; $P = 0.04$). The odds ratios of developing GC for IM in different histological subtypes and stages of GC are shown in Table 5. All odds ratios were significant; a particularly high OR was noted in the intestinal type GC (OR 12.83, 95% CI 4.73–34.83).

### DISCUSSION

In the multistep process of gastric carcinogenesis hypothesized by Correa (1992), microenvironmental factors such as gastric atrophy and IM have been identified. The recent discovery of *H. pylori* as the main causative agent of gastritis leads many researchers to investigate the association of *H. pylori* infection with development of GC. It has been shown that *H. pylori* can increase the prevalence of IM (Rugge et al. 1996). In addition, prospective serological studies have also reported a three- to sixfold increase of risk of GC in *H. pylori*-infected individuals (Forman et al. 1991; Nomura et al. 1991; Parsonnet et al. 1991). In the present study, an OR of 2.43 to contract GC for *H. pylori*-infected subjects was consistent with those previously published. Collectively, these findings support a widely accepted consensus that *H. pylori* is an important environmental carcinogen for GC (Goldstone et al. 1996).

However, the mechanism by which *H. pylori* induces GC remains ill-defined. *H. pylori*-associated chronic gastritis may provide a background for gastric tumorigenesis, in which the intestinal type may be promoted in the atrophic stomach and the diffuse type in the non-atrophic mucosa (Sipponen et al. 1992; Rubin, 1997). However, it seems unlikely that *H. pylori* is solely responsible (Correa, 1992). Recently, it has been shown that gastritis per se rather than its underlying causes may be a more dominant risk factor for GC (Sipponen et al. 1992). Considering the likelihood that *H. pylori*, age and IM may be reciprocal confounding factors for GC, we have used a multiple logistic regression analysis, which disclosed that both *H. pylori* and IM were risk factors for GC. Different odds ratio in developing GC for IM (OR 4.59) or for *H. pylori* infection (OR 2.43) were noted. Our results support the aforementioned hypothesis that IM is a precancerous lesion during the onset of GC (Sipponen et al. 1992; Filipe et al. 1994). This notion is noteworthy because IM is the advanced stage of gastritis and the risk factors for developing IM resemble those for developing GC (Correa et al. 1992; Dobrilla et al. 1994). Diets deficient in fresh fruits and vegetables, combined with a high salt and nitrite intake, are common for both of these two conditions (Correa et al. 1985; Stemmerman et al. 1990; Fuchs and Mayers, 1995). Chronic gastritis due to *H. pylori* also precedes IM and cancer (Sipponen et al. 1992; Goldstone et al. 1996), and accumulating evidence has shown that geographic variation in diet may influence the development of the intestinalized gastritis associated with GC (Stemmermann et al. 1990). Therefore, IM, reflecting the duration and severity of chronic gastritis as a result of the summation of all environmental insults, is a marker more specific for the precancerous lesion of GC. Further studies are needed to ascertain the concomitant relationship between *H. pylori* and other environmental factors, e.g. dietary factors, in the pathogenesis of IM and GC.

Previous studies have noted a variable frequency of IM in GC development (Stemmermann, 1994). One pathoepidemiological study noted that the prevalence of IM was correlated more with intestinal GC than with diffuse GC (Correa et al. 1970). Consistent with this finding, our results also showed that IM was preferentially associated with intestinal GC (OR 12.83) but there was a statistically insignificant difference between the ORs for intestinal and diffuse cancers (OR 3.14). Furthermore, we noted that IM was associated with early GC, as evidenced by a higher OR (6.40) than with advanced GC (OR 3.99), but there was a statistically insignificant difference between them. This finding supports the viewpoint that IM per se is more crucial in the early carcinogenic process, as similarly proposed by Solcia et al (1996), who reported that IM is more frequently found in the gastric mucosae of early rather than advanced cancer.

Three types of IM have been identified, according to the differences in enzyme production, mucus content and presence of Paneth cells (Stemmermann et al. 1994). The different variants of IM may exhibit different risks of GC development (Filipe et al. 1994); thus, type III IM showed stronger risk of GC (OR 39.75) than type II IM (OR 2.03), while type I had a low risk (OR 0.82). The preferential association of type III IM with GC supports the notion that type III IM is most often involved in the pathological transition from dysplasia to carcinoma (Antonioli, 1994).

It is generally believed that IM develops in a background of atrophic gastritis and, in such conditions, pre-existing *H. pylori* colonization of the stomach may be compromised (Forman et al. 1994). Mathialagan et al (1994) have demonstrated that the serological test only possesses a sensitivity of 59% in detecting *H. pylori* infection for patients who have gastric atrophy. Our results in Table 4 revealing a less frequent seropositivity of *H. pylori* in GC associated with type III IM seemed to support the above hypothesis. Similar results have been reported by Masci et al (1996), who showed that the positive rate of *H. pylori* was lower in precancerous lesions, such as dysplasia. It highlighted the possibility that the sensitivity of a serological test might be hampered by the association of gastric atrophy and IM in GC and the role of *H. pylori* might be underestimated.

In conclusion, our data indicate that both *H. pylori* and IM are associated with risk of developing GC and that they might be interrelated. Furthermore, type III IM is a more specific marker for a

© Cancer Research Campaign 1998

premalignant lesion, especially with respect to early and intestinal-type GC.

### ABBREVIATIONS

GC, gastric cancer; IM, intestinal metaplasia; OR, odds ratio; CI, confidence interval

### ACKNOWLEDGEMENTS

This work was supported by grants from the National Science Council (NSC87-2314-B002-235, NSC85-2622-B002-011) and Department of Health, Executive Yuan, Taiwan (DOH86-TD-023, DOH87-TD-1045, DOH87-HR-525).

### REFERENCES

Antonioli DA (1994) Precursors of gastric carcinoma: a critical review with a brief description of early (curable) gastric cancer. *Hum Pathol* **25**: 994–1005

Bechi P, Balzi M, Becciolini A, Mangeri A, Raggi CC, Amorosi A and Dei R (1996) *Helicobacter pylori* and cell proliferation of the gastric mucosa: possible implications for gastric carcinogenesis. *Am J Gastroenterol* **91**: 271–276

Breslow NE and Day NE (1980) *Statistical Methods in Cancer Research*. Vol. 1. *The Analysis of Case–Control Studies*. IARC Scientific Publication no. 31. IARC: Lyon

Caselli M (1996) *Helicobacter pylori*, intestinal metaplasia, and gastric cancer: histopathological point of view. *Am J Gastroenterol* **91**: 1473–1475

Correa P (1992) Human gastric carcinogenesis: a multistep and multifactorial process. *Cancer Res* **52**: 6735–6740

Correa P, Cuello C and Duque E (1970) Carcinoma and intestinal metaplasia of the stomach in Colombian migrants. *J Natl Cancer Inst* **57**: 1027–1035

Correa P, Fontham E, Pickle LW, Chen V, Lin Y and Haenszel W (1985) Dietary determinants of gastric cancer in south Louisiana inhabitants. *J Natl Cancer Inst* **75**: 645–654

Crespi M and Citarda F (1996) *Helicobacter pylori* and gastric cancer: an overrated risk. *Scand J Gastroenterol* **31**: 1041–1046

Dobrilla G, Benvenuti S, Amplatz S and Zancanella L (1994) Chronic gastritis, intestinal metaplasia, dysplasia and *Helicobacter pylori* in gastric cancer: putting the pieces together. *Ital J Gastroenterol* **26**: 449–458

Filipe MI, Munoz N, Matko I, Kato I, Pompe-Kirn V, Jutersek A, Teuchmann S, Benz M and Prijon T (1994) Intestinal metaplasia types and the risk of gastric cancer: a cohort study in Slovenia. *Int J Cancer* **57**: 314–329

Forman D, Newell DG, Fullerton F, Yarnell JW, Stacey AR and Wald N (1991) Association between infection with *Helicobacter pylori* and risk of gastric cancer: evidence from a prospective investigation. *Br Med J* **302**: 1302–1305

Forman D, Webb P and Parsonnet J (1994) *H. pylori* and gastric cancer. *Lancet* **343**: 243–244

Fuchs CS and Mayers RJ (1995) Gastric carcinoma. *N Engl J Med* **333**: 32–41

Goldstone AR, Quirke P and Dixon MF (1996) *Helicobacter pylori* infection and gastric cancer. *J Pathol* **179**: 129–137

Jass JR and Filipe MI (1979) A variant of intestinal metaplasia associated with gastric carcinoma: a histochemical study. *Histopathology* **3**: 191–199

Lauren P (1965) The two histological main types of gastric carcinoma diffuse and so-called intestinal type carcinoma. *Acta Pathol Microbiol Scand* **64**: 31–49

Lev R (1965) The mucin histochemistry of normal and neoplastic gastric mucosa. *Lab Invest* **14**: 2080–2100

Lin JT, Wang JT, Wang TH, Wu MS, Lee TK and Chen CJ (1993) *Helicobacter pylori* infection in a randomly selected population, healthy volunteers, and patients with gastric ulcer and gastric adenocarcinoma: a seroprevalence study in Taiwan. *Scand J Gastroenterol* **28**: 1067–1072

Masci E, Viale E, Freschi M, Porcellati MDM and Tittobello MOA (1996) Precancerous gastric lesions and *Helicobacter pylori*. *Hepatogastroenterology* **43**: 854–858

Mathialogan R, Loizou S, Beales ILP, Scunes D and Calam J (1994) Who gets false-negative *H. pylori* (Hp) Elisa results? *Gut* **35** (suppl. 5): s1

Murakami T (1971) Pathomorphological diagnosis: definition and gross classification of early gastric cancer. *Gann Monogr* **11**: 53–55

Nomura A, Grove JS, Stemmermann GN and Severson RK (1991) *Helicobacter pylori* infection and gastric carcinoma in a population of Japanese-Americans in Hawaii. *N Engl J Med* **325**: 1132–1136

Parkin DM, Pisani P and Fearly J (1993) Estimates of worldwide incidence of eighteen major cancers in 1985. *Int J Cancer* **54**: 594–606

Parsonnet J, Friedman GD, Vandersteen DP, Chang Y, Vogelman JH, Orentreich N and Sibley RK (1991) *Helicobacter pylori* infection and the risk of gastric cancer. *N Engl J Med* **286**: 279–284

Rubin CE (1997) Are there three types of *Helicobacter pylori* gastritis? *Gastroenterology* **112**: 2108–2110

Rugge M, Cassaro M, Leandro G, Baffa R, Avellini C, Bufo P, Stracca V, Battaglia G, Fabiano A, Guerini A and Di Mario F (1996) *Helicobacter pylori* in promotion of gastric carcinogenesis. *Dig Dis Sci* **41**: 950–955

Sipponen P, Kosunen TU, Valle J, Riihel M and Sepp K (1992) *Helicobacter pylori* and chronic gastritis in gastric cancer. *J Clin Pathol* **45**: 319–323

Sobala GM, Schorah CJ, Shires S, Lynch DA, Gallacher B, Dixon MF and Axon AT (1993) Effect of eradication of *Helicobacter pylori* on gastric ascorbic acid concentrations. *Gut* **34**: 1038–1041

Solcia E, Fiocca R, Luinetti O, Villani L, Padovan L, Calistri D, Ranzani GN, Chiaravalli A and Capella C (1996) Intestinal and diffuse gastric cancers arise in a different background of *Helicobacter pylori* gastritis through different gene involvement. *Am J Surg Pathol* **20** (suppl. 1): s8–s22

Stemmermann GN (1994) Intestinal metaplasia of the stomach: a status report. *Cancer* **74**: 556–564

Stemmerman GN, Nomura AMY, Chyou PH and Hankin J (1990) Impact of diet and smoking on risk of developing intestinal metaplasia of the stomach. *Dig Dis Sci* **35**: 432–438

Turani H, Lurie B, Chaimoff C and Kessler E (1986) The diagnostic significance of sulfated acid mucin content in gastric intestinal metaplasia with early gastric cancer. *Am J Gastroenterol* **81**: 343–345

Wu MS, Lin JT, Lee WJ, Yu SC and Wang TH (1994) Gastric cancer in Taiwan. *J Formosan Med Assoc* **93** (suppl. 1): s77–s89

© Cancer Research Campaign 1998


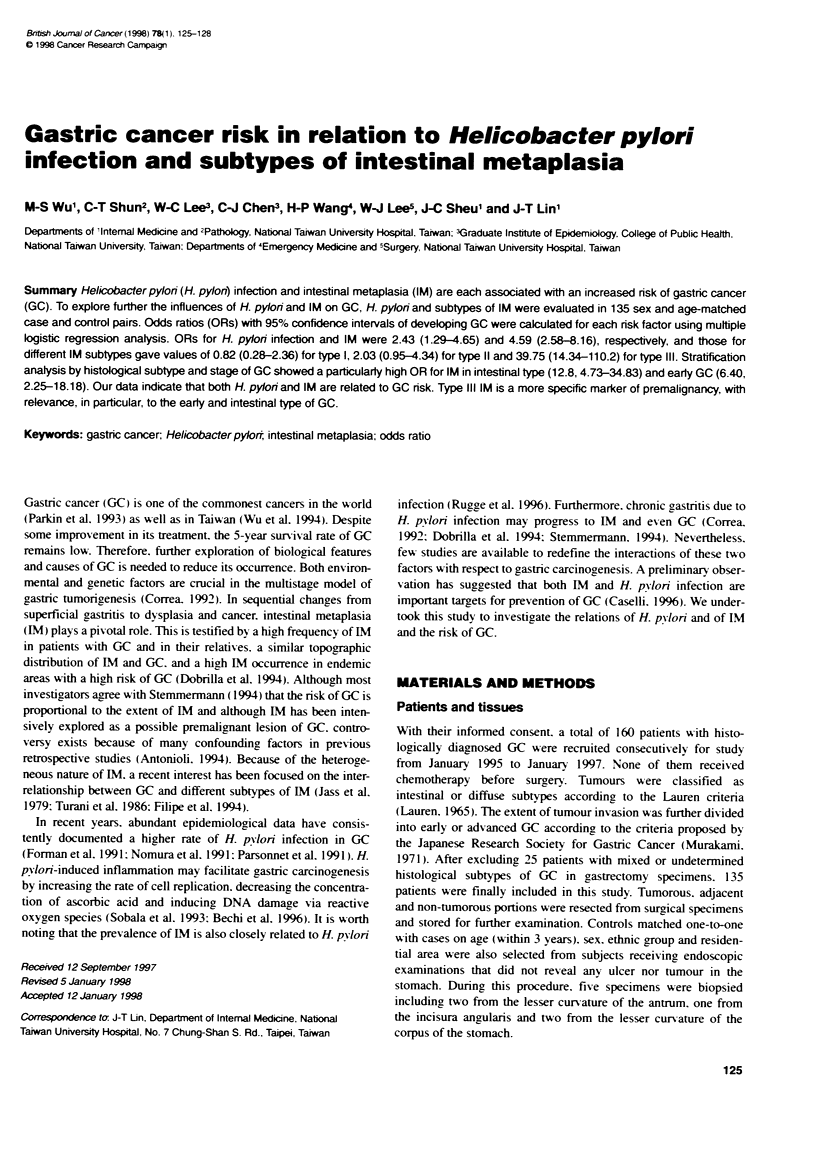

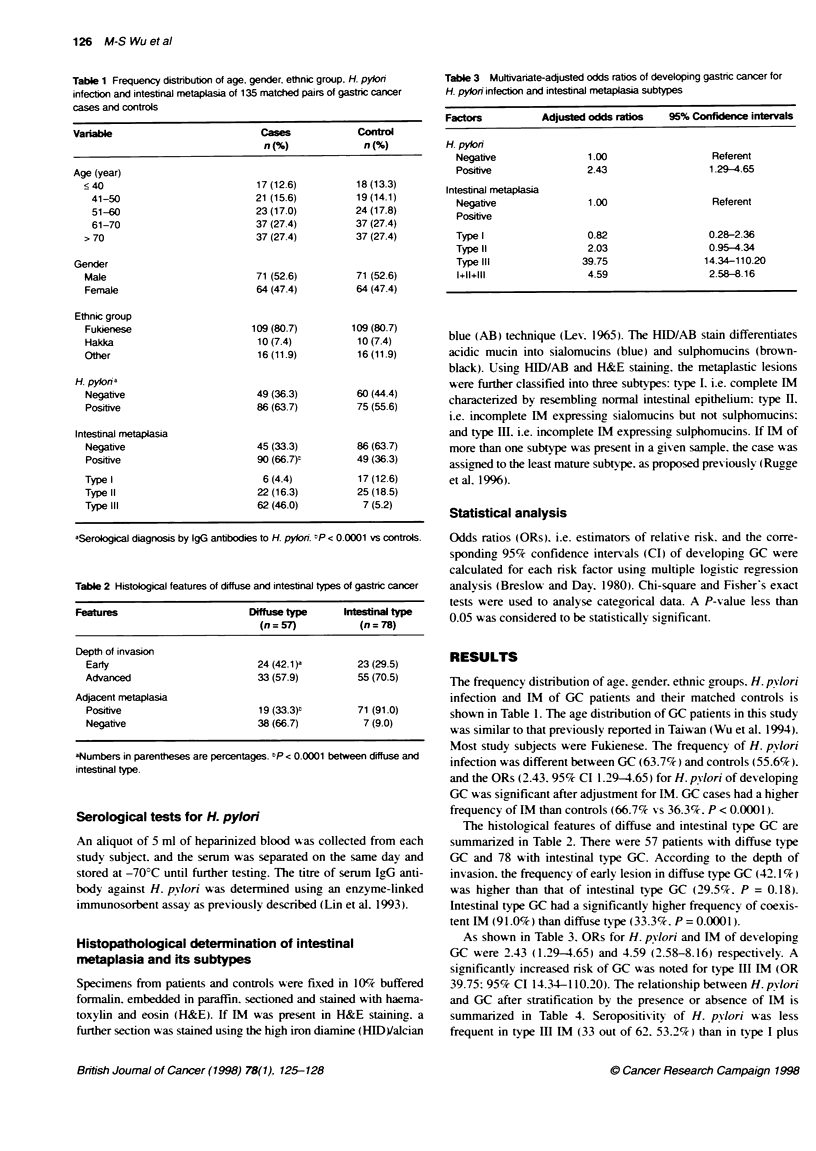

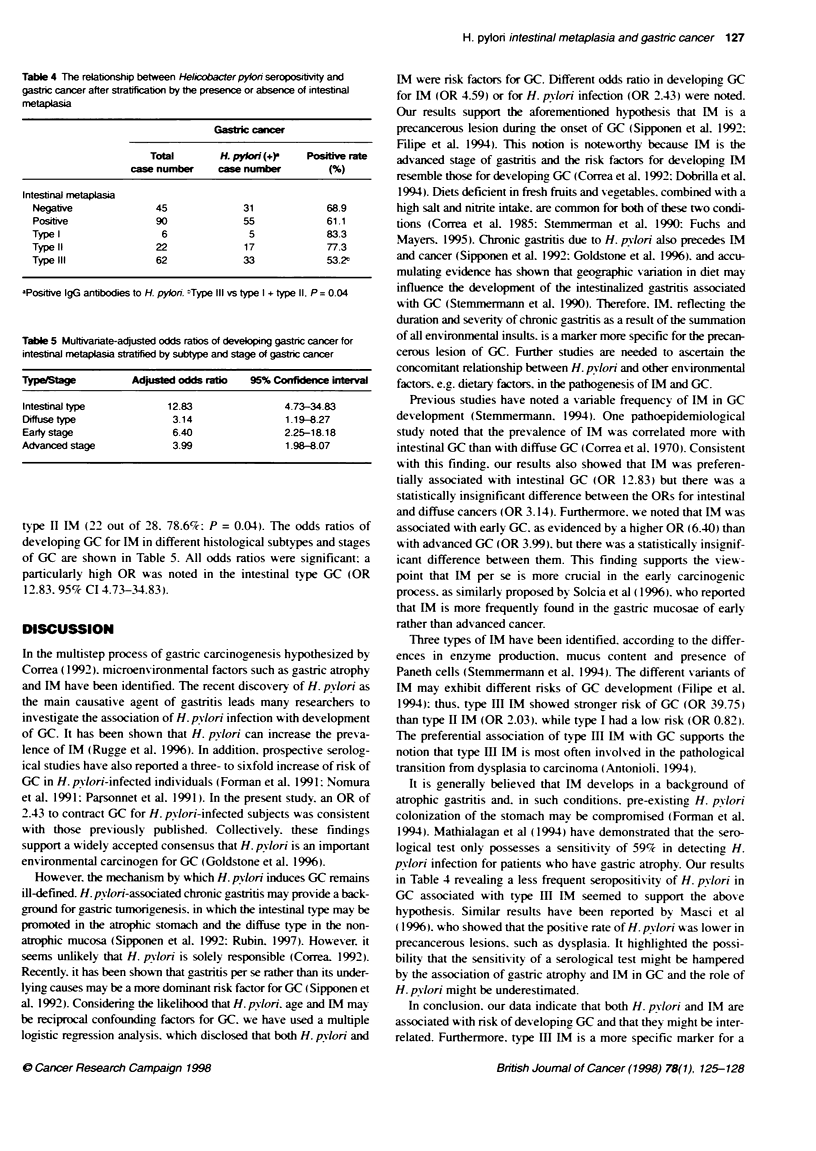

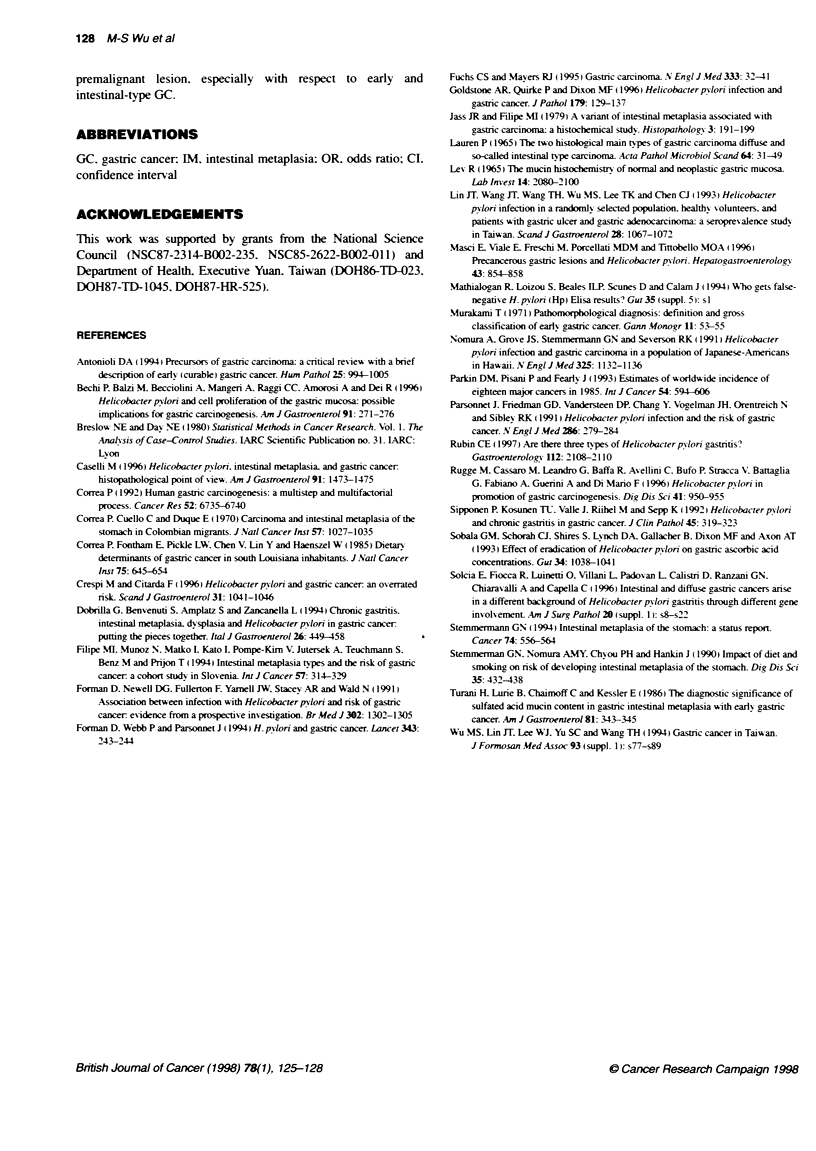


## References

[OCR_00405] Antonioli D. A. (1994). Precursors of gastric carcinoma: a critical review with a brief description of early (curable) gastric cancer.. Hum Pathol.

[OCR_00409] Bechi P., Balzi M., Becciolini A., Maugeri A., Raggi C. C., Amorosi A., Dei R. (1996). Helicobacter pylori and cell proliferation of the gastric mucosa: possible implications for gastric carcinogenesis.. Am J Gastroenterol.

[OCR_00417] Caselli M. (1996). Helicobacter pylori, intestinal metaplasia, and gastric cancer: histopathological point of view.. Am J Gastroenterol.

[OCR_00431] Correa P., Fontham E., Pickle L. W., Chen V., Lin Y. P., Haenszel W. (1985). Dietary determinants of gastric cancer in south Louisiana inhabitants.. J Natl Cancer Inst.

[OCR_00423] Correa P. (1992). Human gastric carcinogenesis: a multistep and multifactorial process--First American Cancer Society Award Lecture on Cancer Epidemiology and Prevention.. Cancer Res.

[OCR_00436] Crespi M., Citarda F. (1996). Helicobacter pylori and gastric cancer: an overrated risk?. Scand J Gastroenterol.

[OCR_00440] Dobrilla G., Benvenuti S., Amplatz S., Zancanella L. (1994). Chronic gastritis, intestinal metaplasia, dysplasia and Helicobacter pylori in gastric cancer: putting the pieces together.. Ital J Gastroenterol.

[OCR_00445] Filipe M. I., Muñoz N., Matko I., Kato I., Pompe-Kirn V., Jutersek A., Teuchmann S., Benz M., Prijon T. (1994). Intestinal metaplasia types and the risk of gastric cancer: a cohort study in Slovenia.. Int J Cancer.

[OCR_00450] Forman D., Newell D. G., Fullerton F., Yarnell J. W., Stacey A. R., Wald N., Sitas F. (1991). Association between infection with Helicobacter pylori and risk of gastric cancer: evidence from a prospective investigation.. BMJ.

[OCR_00456] Forman D., Webb P., Parsonnet J. (1994). H pylori and gastric cancer.. Lancet.

[OCR_00466] Jass J. R., Filipe M. I. (1979). A variant of intestinal metaplasia associated with gastric carcinoma: a histochemical study.. Histopathology.

[OCR_00470] LAUREN P. (1965). THE TWO HISTOLOGICAL MAIN TYPES OF GASTRIC CARCINOMA: DIFFUSE AND SO-CALLED INTESTINAL-TYPE CARCINOMA. AN ATTEMPT AT A HISTO-CLINICAL CLASSIFICATION.. Acta Pathol Microbiol Scand.

[OCR_00473] Lev R. (1965). The mucin histochemistry of normal and neoplastic gastric mucosa.. Lab Invest.

[OCR_00477] Lin J. T., Wang J. T., Wang T. H., Wu M. S., Lee T. K., Chen C. J. (1993). Helicobacter pylori infection in a randomly selected population, healthy volunteers, and patients with gastric ulcer and gastric adenocarcinoma. A seroprevalence study in Taiwan.. Scand J Gastroenterol.

[OCR_00486] Masci E., Viale E., Freschi M., Porcellati M., Tittobello A. (1996). Precancerous gastric lesions and Helicobacter pylori.. Hepatogastroenterology.

[OCR_00497] Nomura A., Stemmermann G. N., Chyou P. H., Kato I., Perez-Perez G. I., Blaser M. J. (1991). Helicobacter pylori infection and gastric carcinoma among Japanese Americans in Hawaii.. N Engl J Med.

[OCR_00502] Parkin D. M., Pisani P., Ferlay J. (1993). Estimates of the worldwide incidence of eighteen major cancers in 1985.. Int J Cancer.

[OCR_00511] Rubin C. E. (1997). Are there three types of Helicobacter pylori gastritis?. Gastroenterology.

[OCR_00515] Rugge M., Cassaro M., Leandro G., Baffa R., Avellini C., Bufo P., Stracca V., Battaglia G., Fabiano A., Guerini A. (1996). Helicobacter pylori in promotion of gastric carcinogenesis.. Dig Dis Sci.

[OCR_00520] Sipponen P., Kosunen T. U., Valle J., Riihelä M., Seppälä K. (1992). Helicobacter pylori infection and chronic gastritis in gastric cancer.. J Clin Pathol.

[OCR_00524] Sobala G. M., Schorah C. J., Shires S., Lynch D. A., Gallacher B., Dixon M. F., Axon A. T. (1993). Effect of eradication of Helicobacter pylori on gastric juice ascorbic acid concentrations.. Gut.

[OCR_00531] Solcia E., Fiocca R., Luinetti O., Villani L., Padovan L., Calistri D., Ranzani G. N., Chiaravalli A., Capella C. (1996). Intestinal and diffuse gastric cancers arise in a different background of Helicobacter pylori gastritis through different gene involvement.. Am J Surg Pathol.

[OCR_00536] Stemmermann G. N. (1994). Intestinal metaplasia of the stomach. A status report.. Cancer.

[OCR_00540] Stemmermann G. N., Nomura A. M., Chyou P. H., Hankin J. (1990). Impact of diet and smoking on risk of developing intestinal metaplasia of the stomach.. Dig Dis Sci.

[OCR_00545] Turani H., Lurie B., Chaimoff C., Kessler E. (1986). The diagnostic significance of sulfated acid mucin content in gastric intestinal metaplasia with early gastric cancer.. Am J Gastroenterol.

[OCR_00550] Wu M. S., Lin J. T., Lee W. J., Yu S. C., Wang T. H. (1994). [Gastric cancer in Taiwan].. J Formos Med Assoc.

